# Therapeutic efficacy of intra-articular injection of human adipose-derived mesenchymal stem cells in a sheep model of knee osteoarthritis

**DOI:** 10.1186/s13287-025-04143-6

**Published:** 2025-01-23

**Authors:** Jigang Lei, Xingyi Chen, Haohao Xie, Yuhao Dai, Zhongjin Chen, Liang Xu

**Affiliations:** Cellular Biopharma (Shanghai) Co., Ltd, Building 3, No.85, Faladi Road, Pudong New Area, Shanghai, 200233 China

**Keywords:** Mesenchymal stem cells, Knee osteoarthritis, Cartilage, Magnetic resonance imaging, Intra-articular injection

## Abstract

**Background:**

Mesenchymal stem cells have great potential for repairing articular cartilage and treating knee osteoarthritis (KOA). Nonetheless, little is known about the efficacy of human adipose-derived mesenchymal stem cells (haMSCs) for KOA in large animal models.

**Methods:**

This study evaluated the therapeutic efficacy of haMSCs in knee articular cartilage repair in a sheep model of KOA. haMSCs were isolated, cultured, and characterized. KOA was surgically induced by anterior cruciate ligament transection and medial meniscectomy, followed by intra-articular injection of saline (negative control group) or haMSCs (haMSC group) into the right knee joint at 6 and 9 weeks after surgery. Sheep were sacrificed 21 weeks after surgery, and samples (whole knee joints, femoral condyles, and tibias) were collected, processed, and analyzed. Changes in knee articular cartilage were assessed by magnetic resonance imaging, micro-computed tomography, macroscopic analysis, histology, and immunohistochemistry.

**Results:**

KOA caused the degeneration of the medial femoral condyle in the sheep model of KOA. Conversely, haMSCs repaired chondral defects and increased the thickness of knee articular cartilage.

**Conclusions:**

These data suggest that the intra-articular injection of haMSCs can effectively repair articular cartilage defects in the knee.

**Supplementary Information:**

The online version contains supplementary material available at 10.1186/s13287-025-04143-6.

## Introduction

Knee osteoarthritis (KOA) is a degenerative disease characterized by progressive knee articular cartilage degeneration and chronic synovial inflammation. KOA is caused by repetitive impact loading. Osteoarthritis (OA) is a leading cause of chronic pain and functional disability worldwide, imposing a significant economic and health burden by decreasing work productivity and the ability to perform activities of daily living. The prevalence of OA increases with age [1–5].

Articular cartilage is a hyaline connective tissue composed predominantly of chondrocytes, which secrete large amounts of type II collagen and proteoglycan and smaller amounts of type VI, IX, XI, and XIV collagen [4]. Articular cartilage has a limited capacity for spontaneous repair because this tissue has low metabolic activity and lacks blood supply, lymphatic drainage, and innervation [4]. Therefore, there is an urgent need to develop minimally invasive, effective, and safe therapeutic strategies for OA.

Mechanical and humoral factors are involved in the pathogenesis of KOA. Nonetheless, the pathogenic mechanisms of KOA are unclear, limiting the development of disease-modifying therapeutics. Moreover, current treatments for KOA have limited efficacy and focus on symptom management rather than articular cartilage repair, and total knee arthroplasty is indicated only to patients with advanced disease [6, 7]. Stem cell therapy is a promising approach for joint tissue regeneration [4]. Mesenchymal stem cells (MSCs) have great potential for treating KOA and chondral defects [8, 9]. Large amounts of MSCs can be harvested from adipose tissue using simple, reproducible, and minimally invasive methods. Furthermore, the quantity and quality of MSCs are significantly higher in adipose tissue than in other tissues. These advantages render adult human adipose tissue an accessible, abundant, and reliable source of MSCs for tissue engineering and regenerative medicine applications [10].

Several animal models have been used to study the onset and progression of OA and determine the efficacy of novel therapies. The selection of appropriate animal models depends on several factors, including animal size, cost, and method of inducing OA [11]. Small animal models are commonly used to analyze the pathophysiology of cartilage degeneration. The advantages of these models include low cost, ease of handling, and high availability. Nonetheless, small animals have a small amount of synovial fluid, which is difficult to collect. Mouse and rabbit models are unsuitable for assessing cartilage repair because repair occurs spontaneously in these models [12, 13]. Moreover, there are marked differences in biomechanical loading and cartilage anatomy between mice and humans.

The advantages of large animal models of OA include anatomic similarity to humans (cartilage thickness and joint size), better suitability for long-term follow-up, high prevalence of naturally occurring primary idiopathic and secondary OA, and capability to perform diagnostic imaging, arthroscopic interventions, synovial fluid collection, and postoperative management. Therefore, these models can generate more clinically relevant data and are usually required for regulatory approval. The disadvantages of these models include high cost, difficulty of handling, longer time to maturity, slower disease progression, and ethical considerations [4, 11, 12, 14]. Another disadvantage of using goats and sheep is that they are not prone to spontaneous OA and thus only serve as models of OA induced surgically by acute joint injury [11, 14].

Little is known about the efficacy of human adipose-derived MSCs (haMSCs) in large animal models of KOA. This study assessed the therapeutic efficacy of haMSCs in a sheep model of KOA surgically induced by anterior cruciate ligament transection (ACLT) and medial meniscectomy (MM).

## Materials and methods

### Isolation and culture of haMSCs

haMSCs were isolated and cultured as described previously [15]. Briefly, haMSCs were isolated from lipoaspirates of healthy young adult donors who gave written informed consent. haMSCs were cultured in T75 cell culture flasks with α-minimum essential medium (α-MEM, C12571500BT, Gibco, USA) supplemented with 5% EliteGro (EPAGMP-500, EliteCell, USA) in a humidified incubator at 37 °C with 5% CO_2_ for 24 h. Detached cells were removed. The remaining cells were maintained in α-MEM and defined as passage 0 (P0). The medium was changed every 2 days. Cells were cultured to 80% confluence. Then, adherent cells were trypsinized and sub-cultured. haMSCs at passage 7 (P7) were harvested for further analysis.

### Flow cytometric analysis

haMSCs (1.0 × 10^6^ cells) at P7 were resuspended in 100 µL of PBS and incubated with 1 µg of phycoerythrin-conjugated, allophycocyanin-conjugated, fluorescein isothiocyanate-conjugated, or peridinin-chlorophyll-protein-Cy5.5-conjugated anti-human monoclonal antibodies for 30 min at 4 °C in the dark [16]. The following antibodies (all from Biolegend, USA) were used: CD73 (#344016), CD90 (#328118), CD105 (#800508), CD14 (#982502), CD45 (#304008), and HLA-DR (#307603). haMSCs were washed with cold 1×PBS and analyzed using a flow cytometer (EPICS XL, Beckman Coulter, Palo Alto, CA, USA).

### Trilineage differentiation of haMSCs in vitro

Trilineage differentiation of haMSCs was performed as described previously [15]. For adipogenic and osteogenic differentiation, haMSCs at P7 were seeded onto 12-well plates at 1.0 × 10^4^ cells and 5.0 × 10^3^ cells per cm^2^, respectively, and cultured in a humidified incubator at 37 °C with 5% CO_2_ until 90% confluence. The StemPro^®^ adipogenic or osteogenic differentiation medium (A1007201, A1007001, Gibco, USA) was added to the culture plates, followed by incubation at 37 °C for 2 to 4 weeks. For chondrogenic differentiation, 1.6 × 10^7^ cells were resuspended in 1 mL of chondrogenic differentiation medium (A1007101, Gibco, USA), centrifuged at 300*g* for 5 min, and cultured in conical tubes for 4 weeks at 37 °C. Samples were fixed in 4% paraformaldehyde for 30 min. The induction of adipogenesis and osteogenesis was confirmed by Oil Red O staining (SC-0843, Sciencell, USA) and Alizarin Red staining (SC-0223, Sciencell, USA), respectively. Chondrogenic pellets were cut using a cryostat. Cryosections were stained with Alcian blue (SC-8348, Sciencell, USA).

### Animals and treatments

Eleven male small-tailed Han sheep aged 15–18 months, with a mean weight of 75 ± 10 kg, were used in this study. After an adaptation period of at least 14 days, the animals were randomly divided into three groups, with three to five animals per group. The normal group (NG) did not undergo treatment. The negative control (NC) group underwent ACLT and MM in the right knee to induce KOA surgically, followed by the intra-articular injection of 5 mL of sterile saline into the knee joint at 6 weeks (one dose) and 9 weeks (one dose) after surgery. The haMSC-treated group (haMSC) underwent surgery as described above, followed by intra-articular injection of haMSCs (1.0 × 10^8^ cells suspended in 5 mL of sterile saline) into the knee joint at 6 weeks (one dose) and 9 weeks (one dose) after surgery [17]. Before surgery, sheep were intramuscularly injected with antibiotics (ceftriaxone sodium, 50 mg/kg) and atropine (0.05 mg/kg). Anesthesia was maintained by the intramuscular injection of Zoletil (2 mg/kg) and Sumianxin II (0.02 mL/kg). Surgery was performed under general anesthesia and sterile conditions, as described previously [18]. After surgery, sheep were intramuscularly injected with buprenorphine (0.01 mg/kg) and ceftriaxone sodium (50 mg/kg) for 3 consecutive days. The NC and haMSC groups were allowed to walk freely. The animals were euthanized at 21 weeks after surgery by the intramuscular injection of Zoletil (2 mg/kg) and the intravenous injection of potassium chloride solution (2 mmol/kg). Samples (whole knee joints, femoral condyles, and tibias) were collected, processed, and examined. The animal studies adhered to the Animal Research: Reporting of In Vivo Experiments (ARRIVE) guidelines 2.0.

### MRI

Three sheep were randomly selected from each group for MRI. Imaging was performed on a 3.0 T MRI scanner (MAGNETOM Skyra, Siemens Healthcare, Erlangen, Germany) at 21 weeks after surgery. Cartilage repair was evaluated by an investigator blinded to treatment allocation using the magnetic resonance observation of cartilage repair tissue (MOCART) scoring system, as previously described [19].

### Macroscopic analysis and micro-computed tomography (CT) scanning

Macroscopic analysis and micro-CT scans of knee joints were performed to assess the degree of degeneration of the articular cartilage of the femoral condyle and cartilage thickness of the tibial plateau, respectively, as detailed previously [17]. Right femoral condyles were collected and imaged. Cartilage repair was assessed by an observer blinded to treatment allocation using the International Cartilage Research Society (ICRS) criteria [20].

Micro-CT scans of the proximal tibial plateau were performed using the following parameters: voxel size, 45 mm; tube voltage, 70 kVp; tube current, 114 µA; integration time, 200 ms; acquisition time, 15–30 min. Image segmentation was performed using histogram thresholding (threshold value of 30-1000) and the following Gauss filter parameters: sigma, 1.2; support, 2. Average cartilage thickness in segmented images was measured using distance transformation algorithms [21]. CT images were analyzed by an investigator blinded to treatment allocation.

### Histological and immunohistochemical analyses

Femoral condyles were fixed in 4% paraformaldehyde for 24 h. The specimens were washed with distilled water, decalcified in EDTA, dehydrated in a graded ethanol series, dewaxed with dimethylbenzene, and embedded in paraffin. The specimens were serially sectioned at a thickness of 5 μm and used for hematoxylin and eosin (H&E), Safranin O/Fast Green, and immunohistochemical staining. The degree of articular degeneration was graded histologically using Mankin scores [22]. Cartilage thickness was measured by an investigator blinded to treatment allocation as detailed previously [23].

For immunohistochemical staining, tissue sections were incubated with polyclonal rabbit anti-sheep collagen II (Abcam, 1:100) or collagen X (Abcam, 1:100) antibodies at 4 °C overnight, followed by incubation with biotin-conjugated anti-rabbit IgG (Abcam, 1:500) at room temperature for 1 h. Sections were imaged using a light microscope (AxioScope A1, Carl Zeiss MicroImaging GmbH, Germany). The area stained positive for collagen II and the number of chondrocytes expressing collagen X per field were analyzed using ImageJ software (NIH, USA).

### Statistical analysis

Statistical analysis was performed using GraphPad Prism version 10.0 (GraphPad Software Inc., La Jolla, CA). Normally distributed continuous variables were expressed as mean ± standard error of the mean. The significance of differences between means was assessed by one-way analysis of variance followed by Newman-Keuls post-hoc test. P-values of less than 0.05 were considered statistically significant.

## Results

### Characterization of haMSCs in vitro

**Fig. 1:**
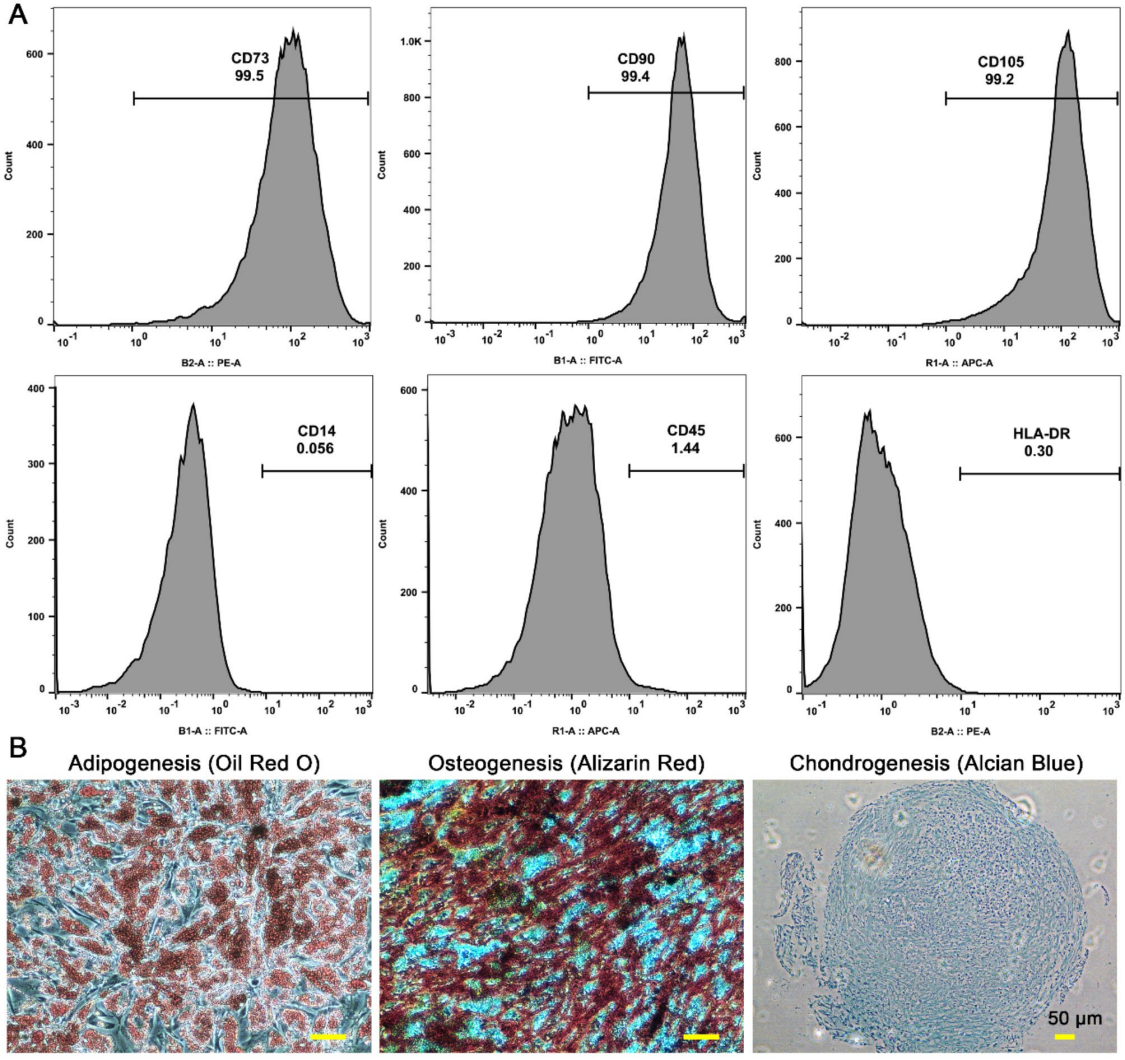
Characterization of human adipose-derived mesenchymal stem cells (haMSCs). **(A)** Flow cytometric analysis of cell surface markers CD73, CD90, CD105, CD14, CD45, and HLA-DR on haMSCs. **(B)** Trilineage differentiation of haMSCs into adipocytes, osteocytes, and chondrocytes based on Oil Red O, Alizarin Red, and Alcian Blue staining. Scale bar: 50 μm

haMSCs were characterized by flow cytometry and histology (trilineage differentiation). Cytometric analysis showed that these cells were positive for CD73, CD90, and CD105 (≥ 95%) and negative for CD14, CD45, and HLA-DR (≤ 2%) (Fig. 1A). Histology showed that haMSCs differentiated into adipocytes, osteocytes, or chondrocytes in the presence of differentiation medium (Fig. 1B). These results demonstrate that isolated cells had phenotypic characteristics of MSCs.

### MRI of changes in knee articular cartilage

**Fig. 2:**
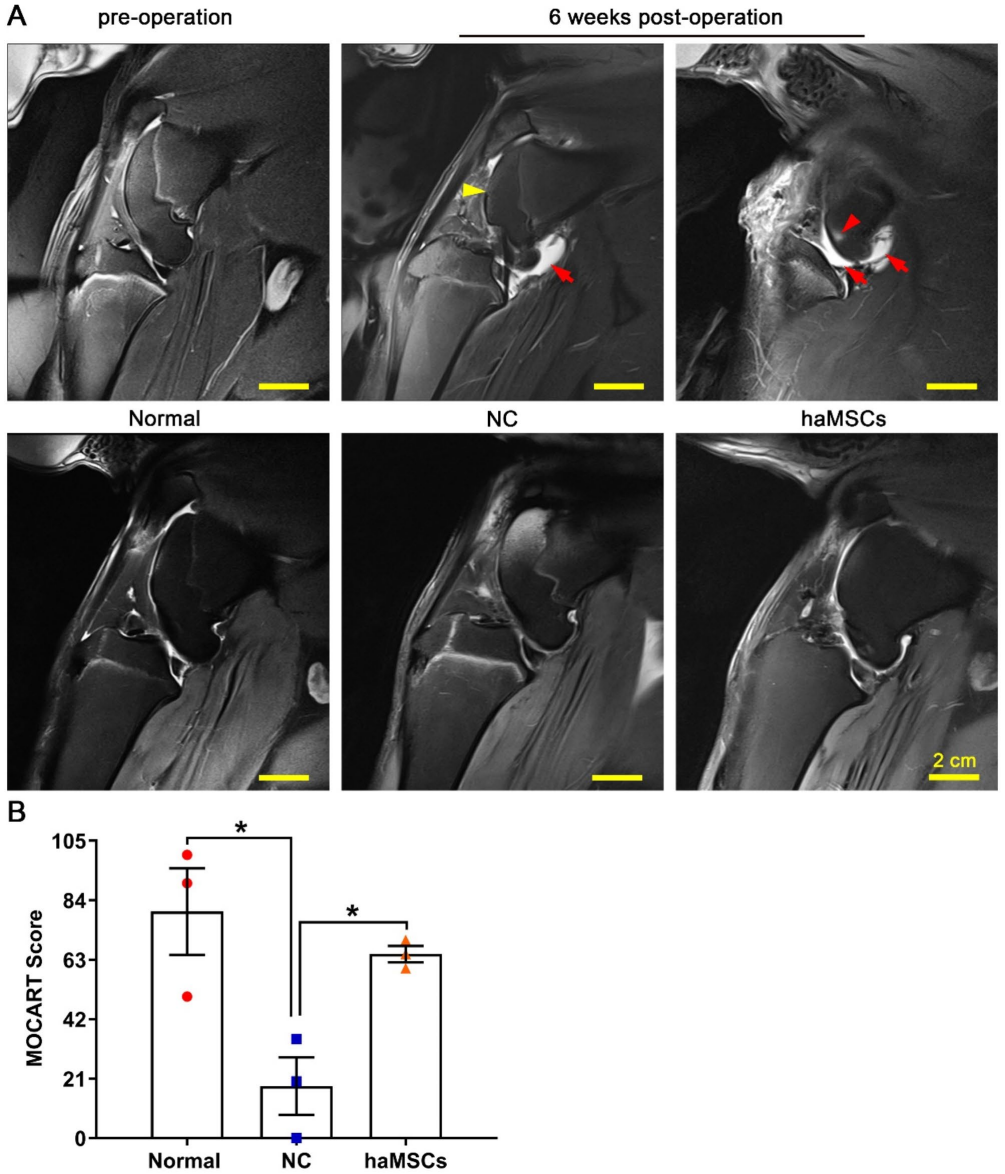
Magnetic resonance imaging (MRI) of knee articular cartilage in normal sheep and in a sheep model of knee osteoarthritis surgically induced by anterior cruciate ligament transection and medial meniscectomy, followed by intra-articular injection of sterile saline (NC) or haMSCs into the right knee joint at 6 weeks (one dose) and 9 weeks (one dose) after surgery. **(A)** MRI analysis of changes in articular cartilage in different groups. The red arrows, yellow arrowhead, and red arrowhead indicate joint effusion, osteophyte formation, and bone marrow edema. Scale bar: 2 cm. **(B)** MOCART scores based on MRI results in different groups (three animals per group). *p < 0.05. Abbreviations: NC, negative control; haMSCs, human adipose-derived mesenchymal stem cells

The therapeutic efficacy of haMSCs on articular cartilage repair was investigated by MRI. At 6 weeks post-surgery, MRI showed joint effusion (red arrows), osteophyte formation (yellow arrowhead), bone marrow edema (red arrowhead), articular cartilage with ill-defined margins, and the absence of the anterior cruciate ligament (ACL) and medial meniscus, indicating the successful creation of the sheep model of KOA (Fig. 2A). haMSCs reduced these effects at 21 weeks post-surgery, evidenced by the finding that the knee cartilage in the haMSC-treated group was continuous, smooth, and intact, similar to cartilage in the NG (Fig. 2A). KOA significantly decreased MOCART scores, while haMSCs reversed this effect (*p* < 0.05) (Fig. 2B). These data indicate that cellular treatment repaired knee cartilage defects.

### haMSCs improve the morphology of femoral condyles

**Fig. 3:**
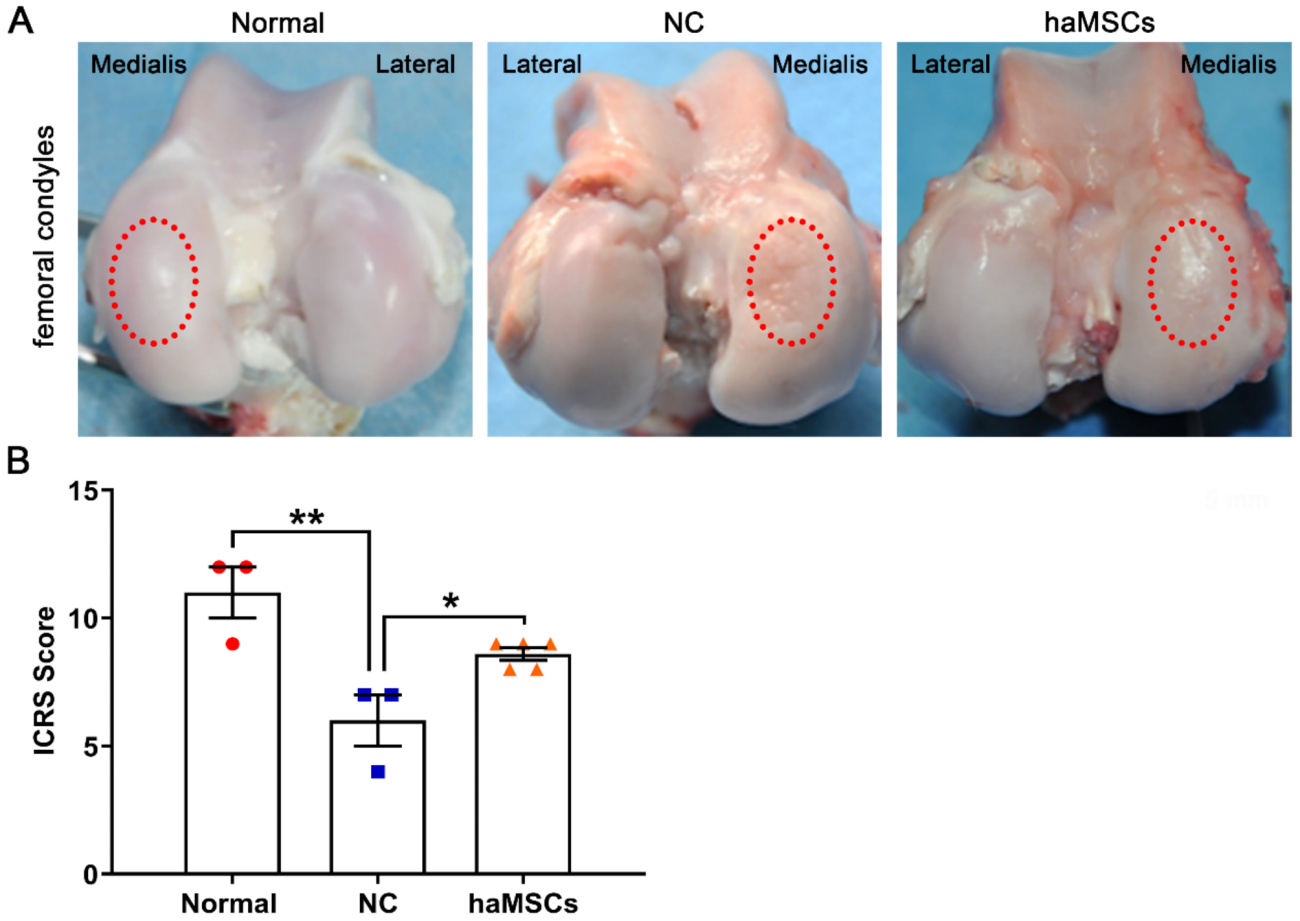
Macroscopic analysis of sheep femoral condyles. **(A)** Macroscopic observation of femoral condyles in normal sheep and in a sheep model of knee osteoarthritis surgically induced by anterior cruciate ligament transection and medial meniscectomy followed by intra-articular injections of sterile saline (NC) or haMSCs into the right knee joint at 6 weeks (one dose) and 9 weeks (one dose) after surgery. The dotted circles show the medial condyle. **(B)** Cartilage repair was assessed using ICRS criteria (three to five animals per group). *p < 0.05, **p < 0.01. Abbreviations: NC, negative control; haMSCs, human adipose-derived mesenchymal stem cells; ICRS, International Cartilage Repair Society

**Fig. 4:**
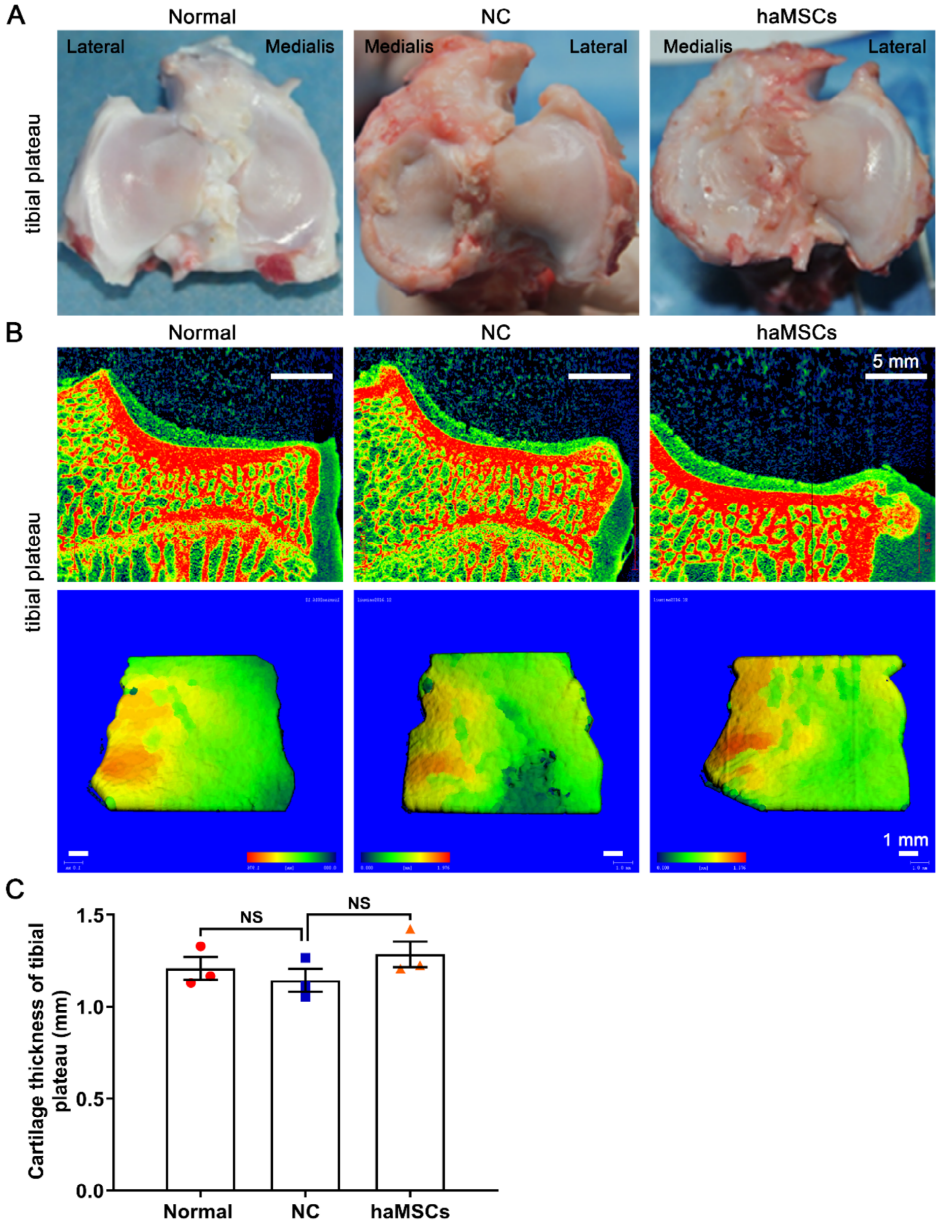
Macroscopic analysis and micro-computed tomography (micro-CT) scans of sheep tibial plateau. **(A)** Macroscopic analysis of tibial plateaus in normal sheep and sheep who underwent knee osteoarthritis surgically induced by anterior cruciate ligament transection and medial meniscectomy, followed by intra-articular injections of sterile saline (NC) or haMSCs into the right knee joint at 6 weeks (one dose) and 9 weeks (one dose) after surgery. **(B)** Micro-CT scans of the tibial plateau in experimental and control groups. **(C)** Measurement of cartilage thickness in the tibial plateau of the experimental and control groups (three animals per group). Abbreviations: NC, negative control; haMSCs, human adipose-derived mesenchymal stem cells; NS, not significant

The therapeutic efficacy of haMSCs on knee cartilage repair was evaluated by the macroscopic analysis of the articular cartilage of the femoral condyle and tibial plateau. The results showed that KOA caused the degeneration of the medial femoral condyle, while haMSCs reduced the degeneration (Fig. 3A). KOA significantly decreased ICRS scores (*p* < 0.01), while haMSCs reduced this effect (*p* < 0.05) (Fig. 3B). Nonetheless, macroscopic analysis showed that haMSCs did not improve tibial plateau morphology to a significant extent (Fig. 4A).

Micro-CT scans showed no notable changes in the microstructure of tibial plateau cartilage in the three groups (Fig. 4B). Further, there were no significant between-group differences in tibial plateau cartilage thickness (Fig. 4C). These findings indicate that two intra-articular injections of haMSCs can effectively repair knee articular cartilage defects in the sheep model of KOA.

### haMSCs promote knee cartilage regeneration

**Fig. 5:**
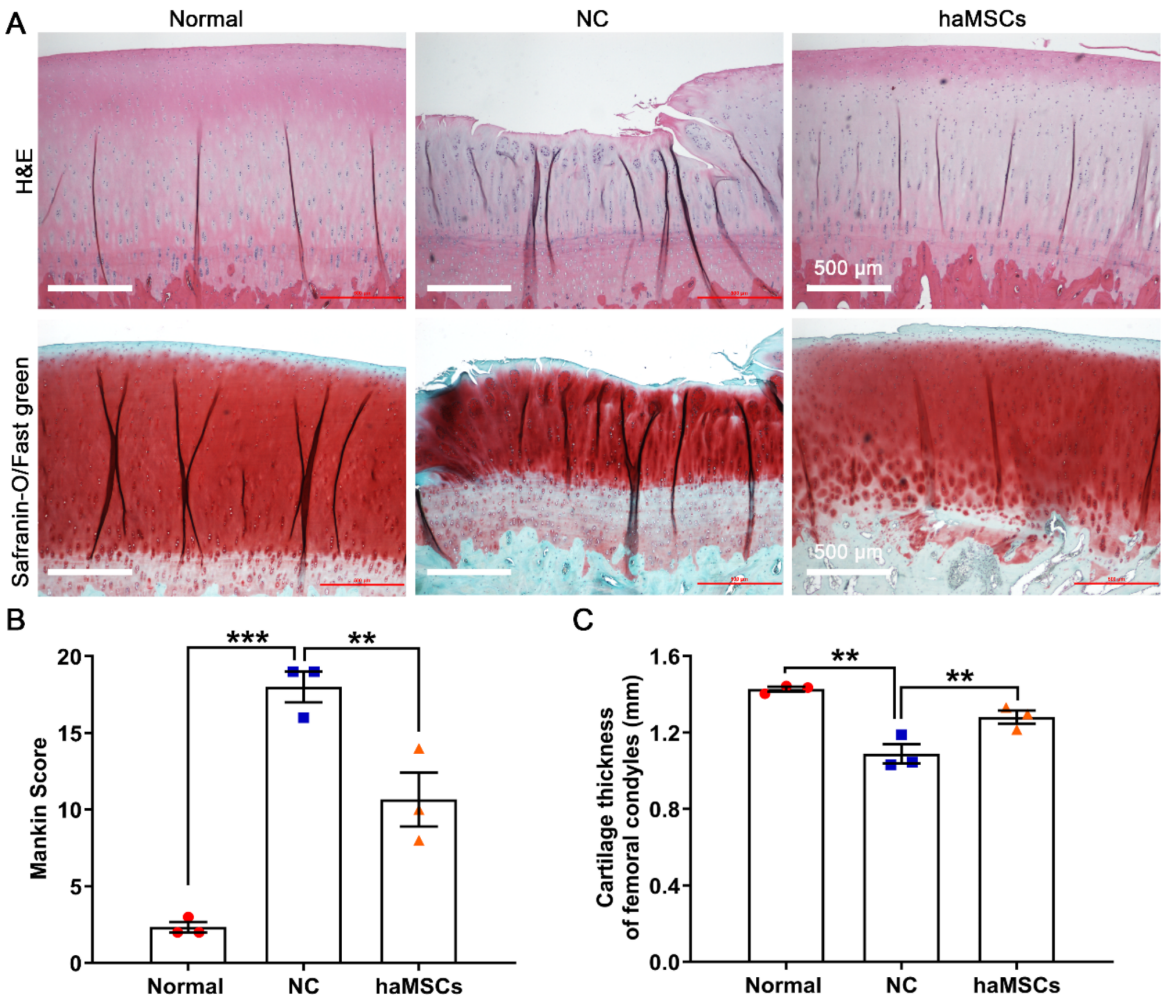
Histological analysis of knee articular cartilage in sheep. **(A)** H&E staining and Safranin O/Fast Green staining of cartilage sections of normal sheep and sheep who underwent knee osteoarthritis surgically induced by anterior cruciate ligament transection and medial meniscectomy, followed by intra-articular injections of sterile saline (NC) or haMSCs into the right knee joint at 6 weeks (one dose) and 9 weeks (one dose) after surgery. **(B)** Analysis of the degree of cartilage degeneration using the Mankin scoring system in the experimental and control groups (three animals per group). **p < 0.01, ***p < 0.001. **(C)** Measurement of femoral condyle cartilage thickness (three animals per group). **p < 0.01. Abbreviations: H&E, hematoxylin and eosin; NC, negative control; haMSCs, human adipose-derived mesenchymal stem cells

The effects of haMSCs on cartilage repair were assessed by histological analysis of femoral condyle cartilage at 21 weeks post-surgery. H&E and Safranin O/Fast Green staining revealed that the articular cartilage surface was rough and heterogeneous in the NC group. haMSCs attenuated these pathological changes, and the cartilage surface was smooth and continuous, with a high concentration of proteoglycans (Fig. 5A). KOA significantly increased Mankin scores (*p* < 0.001) and decreased femoral cartilage thickness (*p* < 0.01); haMSC treatment attenuated these effects (*p* < 0.01) (Fig. 5B, C). These results demonstrate that haMSCs promote articular cartilage regeneration in the sheep model of KOA.

### haMSCs reverse the changes in the cartilage matrix induced by KOA

**Fig. 6:**
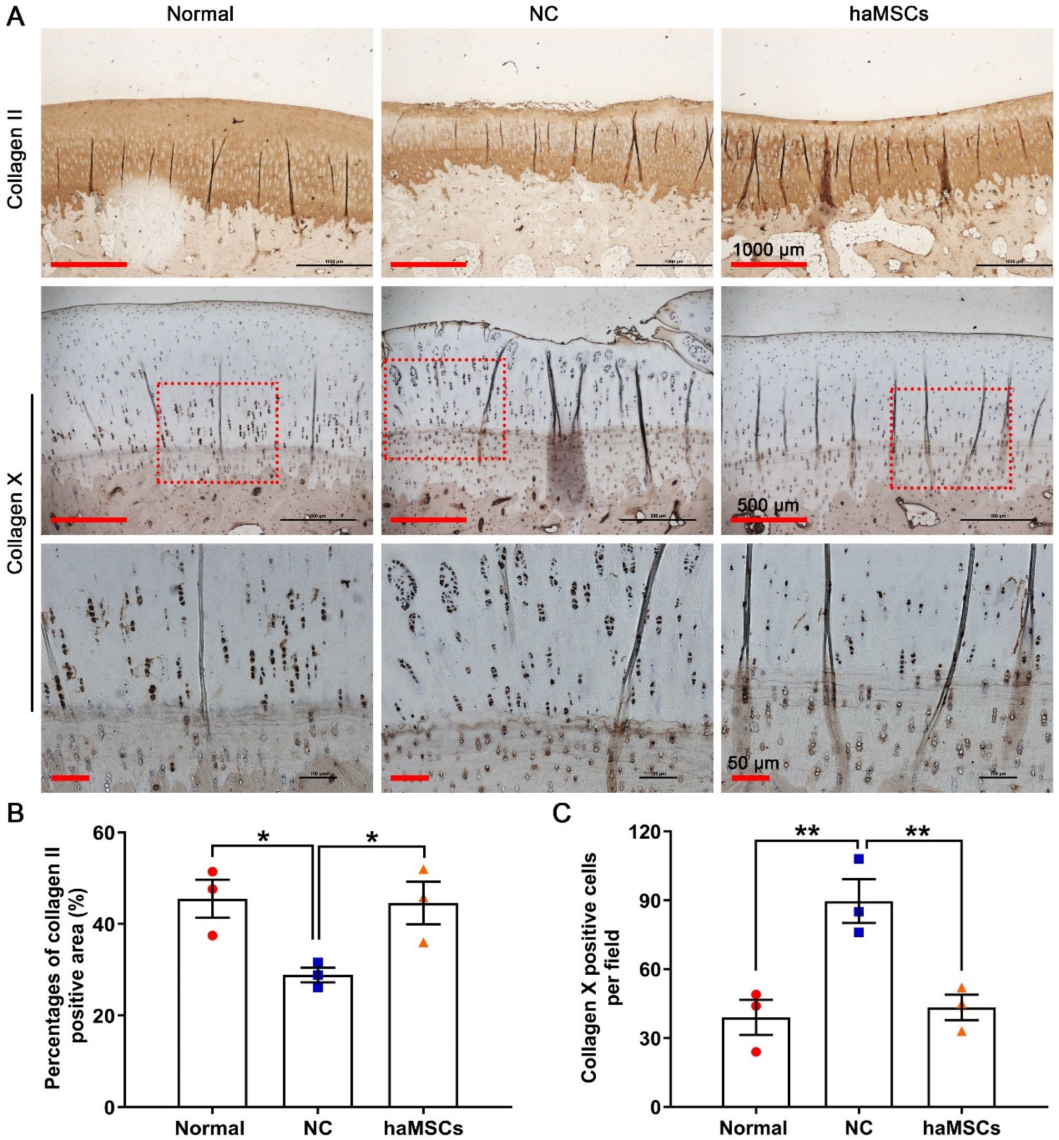
Immunohistochemical expression of type II and X collagen in femoral condyle cartilage. **(A)** Immunohistochemical expression of type II and X collagen in the femoral articular cartilage of normal sheep and in a sheep model of knee osteoarthritis surgically induced by anterior cruciate ligament transection and medial meniscectomy, followed by the intra-articular injection of sterile saline (NC) or haMSCs into the right knee joint at 6 weeks (one dose) and 9 weeks (one dose) after surgery. Scale bars: 1000, 500, and 50 μm. **(B)** Area stained positive for type II collagen (three animals per group). *p < 0.05. **(C)** Number of hypertrophic chondrocytes expressing type X collagen per field. The red squares are magnified images (three animals per group). **p < 0.01. Abbreviations: NC, negative control; haMSCs, human adipose-derived mesenchymal stem cells

The effects of haMSCs on the articular cartilage matrix were evaluated by determining the immunohistochemical expression of collagen II and collagen X at 21 weeks post-surgery. KOA markedly decreased the area stained positive for type II collagen (*p* < 0.05) and increased the number of hypertrophic chondrocytes expressing type X collagen in the deep zone (*p* < 0.01); haMSCs reversed these effects (Fig. 6A-C). These findings suggest that cellular treatment can promote femoral cartilage repair in the sheep model of KOA.

## Discussion

Given that repeated injections of umbilical cord-derived MSCs were better than a single injection for WOMAC and pain scores in patients with KOA [24], we performed two intra-articular injections of haMSCs in our animal model of KOA at weeks 6 and 9 after surgery. We found that cartilage defects were more evident in the medial femoral condyle than in the lateral condyle. ACL deficiency causes maximum erosion in the medial compartment without notable changes in the lateral compartment because the former supports more mechanical load than the latter [25, 26]. Our results also suggest that the medial condyle is more prone to wear than the lateral condyle. Additionally, there were no changes in the thickness of the tibial plateau cartilage in the study groups. Articular cartilage wear is higher on the femoral side than on the tibial side because a larger area of the cartilage is used on the femoral side during knee motion [26].

Chondrocyte hypertrophy is a hallmark of OA [27]. Hypertrophic chondrocytes can cause cartilage matrix degradation by secreting inflammatory and metabolic markers, including collagen X and MMP13 [28]. Therefore, chondrocyte hypertrophy inhibition is a promising therapeutic target for OA. Consistent with these results, we found that at 21 weeks post-surgery, KOA caused cartilage defects in the medial femoral condyle, decreased the expression of type II collagen in the cartilage matrix, and increased the number of hypertrophic chondrocytes expressing type X collagen in the deep zone. haMSCs attenuated these effects, indicating that cellular treatment reduced KOA progression by inhibiting chondrocyte hypertrophy. Furthermore, we previously showed that multiple intra-articular injections of haMSCs promoted articular cartilage regeneration in a rabbit model of OA [15]. In line with these findings, we showed that two intra-articular injections of haMSCs reduced KOA progression by promoting knee articular cartilage repair and increasing cartilage thickness in a sheep model of KOA.

Aging affects the ability of chondrocytes to maintain and restore articular cartilage. Aging is associated with fewer chondrocytes, chondrocyte senescence, and chondrocyte apoptosis, increasing the risk of articular cartilage degeneration [4]. Therefore, aging decreases the therapeutic efficacy of MSCs in OA [29]. Participation in sports involving high axial and torsional loading increases the risk of joint injuries and degeneration [2]. The sheep model of KOA does not entirely mimic aging-related KOA but mimics post-traumatic KOA. Many preclinical studies used animal models of surgically induced KOA. Nonetheless, animal models cannot accurately mimic the pathology of human aging-related KOA. KOA in animal models of naturally occurring disease progresses slowly. Hence, the duration of disease and treatment is long in these models [30]. Our study used an animal model of post-traumatic KOA with or without haMSC treatment. This model can mimic some aspects of human pathology because post-traumatic KOA occurs in humans. However, further studies are needed to assess whether haMSCs can treat aging-related KOA.

The effects of haMSCs on cartilage repair were assessed using a sheep model of KOA induced by ACLT and MM. The findings showed that haMSCs promoted knee articular cartilage repair in this model. Consistent with this finding, our pilot studies and clinical trials showed that intra-articular injections of haMSCs reduced pain scores and improved cartilage regeneration and physical function in patients with KOA [10, 31, 32]. Other studies suggest that exogenous MSCs do not differentiate into chondrocytes but participate in cartilage repair by regulating local inflammation and paracrine signaling [33, 34]. Further, MSCs secrete enzymes, chemokines, cytokines, growth factors, and microRNAs [34, 35].

Growth factors promote cartilage repair by stimulating the proliferation of endogenous joint-resident MSCs, which differentiate into chondrocytes and induce the formation of cartilage-associated extracellular matrix [36–42]. Consistent with these results, we identified and quantified various growth factors in the conditioned medium of haMSCs, including vascular endothelial growth factor, platelet-derived growth factor, hepatocyte growth factor, and fibroblast growth factor 2 (FGF-2) (Supplementary Table 1). FGF-2 promotes the repair of full-thickness defects of articular cartilage by activating the proliferation and migration of MSCs [43].

MSC-derived exosomes contain growth factors and microRNAs that reduce inflammation and induce tissue repair [34, 44–46], demonstrating that haMSC-derived exosomes can potentially repair KOA-induced cartilage defects. We previously showed that hsa-miR-92a-3p in haMSC-derived exosomes enhanced chondrogenesis and suppressed cartilage degradation by targeting Wnt5a [44, 47]. Our results suggest that two intra-articular injections of haMSCs can repair chondral defects in the medial femoral condyle in a sheep model of KOA.

Many pilot studies and clinical trials have assessed the efficacy of human stem cell-based therapies for KOA. Nonetheless, few clinical studies have evaluated the effects of these therapies on cartilage structure, and few preclinical studies have evaluated the effects of these therapies in large animal models of KOA. Our results showed that haMSCs improved knee cartilage structure in sheep, supporting the clinical application of haMSCs. Additionally, haMSCs are routinely cultured for GMP production and have a good safety profile. We intend to use a 3D culture system to increase production scale and efficiency and decrease production costs. Although MSCs are currently used for treating KOA, several treatment parameters need to be determined in large animal models of KOA, including the optimal dose and frequency, the interval between injections, and the efficacy of different MSC passages.

This study has limitations. First, functional outcomes were not assessed in our model. Second, repeated intra-articular injections of haMSCs in our model may cause immune reactions. Although we previously demonstrated the safety of haMSC-based therapies for KOA [10, 31, 32], potential side effects and risks should be evaluated. Further, combination therapy with MSCs and bioactive compounds may have better therapeutic effects on KOA than MSCs alone [48–51]. For instance, we showed that intra-articular injection of sheep autologous or allogeneic adipose MSCs combined with hyaluronic acid ameliorated osteoarthritis in sheep [17, 52], demonstrating the potential applicability of combination therapies for cartilage repair in patients with KOA [53]. However, further studies are needed to assess whether combination therapies can improve cartilage regeneration in large animal models of KOA.

## Conclusion

Intra-articular injection of haMSCs can repair articular cartilage defects in the femoral condyle in a sheep model of KOA, demonstrating the potential of MSCs for treating OA.

## Electronic supplementary material

Below is the link to the electronic supplementary material.

Supplementary Material 1: Table 1. Quantification of growth factors in the conditioned medium of haMSCs (in pg/mL).

## Data Availability

All other data are included in the article and its Supplementary Information files or available from the corresponding authors upon reasonable request.

## Abbreviations

KOA: Knee osteoarthritis
haMSCs: human adipose-derived mesenchymal stem cells
OA: Osteoarthritis
ACLT: Anterior cruciate ligament transection
MM: Medial meniscectomy
NG: Normal group
NC: Negative control
MRI: Magnetic resonance imaging
MOCART: Magnetic resonance observation of cartilage repair tissue
micro-CT: micro-computed tomography
ICRS: International Cartilage Research Society
H&E: Hematoxylin and eosin
VEGF: Vascular endothelial growth factor
PDGF: Platelet-derived growth factor
HGF: Hepatocyte growth factor
FGF: Fibroblast growth factor

## References

[1] Sakai R, Cho SK, Jang EJ, Harigai M, Sung YK. International descriptive study for comparison of treatment patterns in patients with knee osteoarthritis between Korea and Japan using claims data. Int J Rheum Dis. 2019;22(11):2052–8. 31692273 10.1111/1756-185X.13711

[2] Buckwalter JA. Sports, joint injury, and posttraumatic osteoarthritis. J Orthop Sports Phys Ther. 2003;33(10):578–88. 14620787 10.2519/jospt.2003.33.10.578

[3] Freitag J, Bates D, Boyd R, Shah K, Barnard A, Huguenin L, Tenen A. Mesenchymal stem cell therapy in the treatment of osteoarthritis: reparative pathways, safety and efficacy. BMC Musculoskelet Disord. 2016;26(17):230. 10.1186/s12891-016-1085-9 PMC4880954 27229856

[4] Thoene M, Bejer-Olenska E, Wojtkiewicz J. The current state of osteoarthritis treatment options using stem cells for regenerative therapy: a review. Int J Mol Sci. 2023;24(10):8925. 37240271 10.3390/ijms24108925 PMC10219560

[5] Steinmetz JD, Culbreth GT, Haile LM, Rafferty Q, Lo J, Fukutaki KG. Global, regional, and national burden of osteoarthritis, 1990–2020 and projections to 2050: a systematic analysis for the global burden of disease study 2021. Lancet Rheumatol. 2023;5(9):e508–22. 37675071 10.1016/S2665-9913(23)00163-7 PMC10477960

[6] Pers YM, Ruiz M, Noël D, Jorgensen C. Mesenchymal stem cells for the management of inflammation in osteoarthritis: state of the art and perspectives. Osteoarthritis Cartilage. 2015;23(11):2027–35. 26521749 10.1016/j.joca.2015.07.004

[7] Zhang S, Hu B, Liu W, Wang P, Lv X, Chen S, Liu H, Shao Z. Articular cartilage regeneration: the role of endogenous mesenchymal stem/progenitor cell recruitment and migration. Semin Arthritis Rheum. 2020;50(2):198–208. 31767195 10.1016/j.semarthrit.2019.11.001

[8] Doyle EC, Wragg NM, Wilson SL. Intraarticular injection of bone marrow-derived mesenchymal stem cells enhances regeneration in knee osteoarthritis. Knee Surg Sports Traumatol Arthrosc. 2020;28(12):3827–42. 32006075 10.1007/s00167-020-05859-z PMC7669782

[9] Maheshwer B, Polc EM, Paul K, Williams BT, Wolfson TS, Yanke A, Verma NN, Cole BJ, Chahla J. Regenerative potential of mesenchymal stem cells for the treatment of knee osteoarthritis and chondral defects: a systematic review and meta-analysis. Arthroscopy. 2021;37(1):362–78. 32497658 10.1016/j.arthro.2020.05.037

[10] Song Y, Du H, Dai C, Zhang L, Li S, Hunter DJ, Lu L, Bao C. Human adipose-derived mesenchymal stem cells for osteoarthritis: a pilot study with long-term follow-up and repeated injections. Regen Med. 2018;13(3):295–307. 29417902 10.2217/rme-2017-0152

[11] Gregory MH, Capito N, Kuroki K, Stoker AM, Cook JL, Sherman SL. A review of translational animal models for knee osteoarthritis. Arthritis. 2012; 2012:764621. 10.1155/2012/764621 PMC3541554 23326663

[12] McCoy AM. Animal models of osteoarthritis: comparisons and key considerations. Vet Pathol. 2015;52(5):803–18. 26063173 10.1177/0300985815588611

[13] Zaki S, Blaker CL, Little CB. OA foundations-experimental models of osteoarthritis. Osteoarthritis Cartilage. 2022;30(3):357–80. 34536528 10.1016/j.joca.2021.03.024

[14] Teeple E, Jay GD, Elsaid KA, Fleming BC. Animal models of osteoarthritis: challenges of model selection and analysis. AAPS J. 2013;15(2):438–46. 23329424 10.1208/s12248-013-9454-x PMC3675748

[15] Wang W, He N, Feng C, Liu V, Zhang L, Wang F, He J, Zhu T, Wang S, Qiao W, Li S, Zhou G, Zhang L, Dai C, Cao W. Human adipose-derived mesenchymal progenitor cells engraft into rabbit articular cartilage. Int J Mol Sci. 2015;16(6):12076–91. 26023716 10.3390/ijms160612076 PMC4490430

[16] Lei J, Xu Z, Li S, Li M, Wang Z, Li P, Wang J, Chen Y, Song X, Ren C, Shen M, Dai C. Human adipose, placenta, and umbilical cord-derived mesenchymal stem cells ameliorate imiquimod-induced psoriatic mice via reducing T cells infiltration. Biocell. 2021;45(3):537–46.

[17] Lv X, He J, Zhang X, Luo X, He N, Sun Z, Xia H, Liu V, Zhang L, Lin X, Lin L, Yin H, Jiang D, Cao W, Wang R, Zhou G, Wang W. Comparative efficacy of autologous stromal vascular fraction and autologous adipose-derived mesenchymal stem cells combined with hyaluronic acid for the treatment of sheep osteoarthritis. Cell Transpl. 2018;27(7):1111–25. 10.1177/0963689718773333 PMC6158543 29909687

[18] Mokbel AN, El Tookhy OS, Shamaa AA, Rashed LA, Sabry D, El Sayed AM. Homing and reparative effect of intra-articular injection of autologus mesenchymal stem cells in osteoarthritic animal model. BMC Musculoskelet Disord. 2011;15(12):259. 10.1186/1471-2474-12-259 PMC3232438 22085445

[19] Marlovits S, Striessnig G, Resinger CT, Aldrian SM, Vecsei V, Imhof H, Trattnig S. Definition of pertinent parameters for the evaluation of articular cartilage repair tissue with high-resolution magnetic resonance imaging. Eur J Radiol. 2004;52(3):310–9. 15544911 10.1016/j.ejrad.2004.03.014

[20] Brittberg M, Winalski CS. Evaluation of cartilage injuries and repair. J Bone Joint Surg Am. 2003;85(A Suppl):58–69. 12721346 10.2106/00004623-200300002-00008

[21] Kotwal N, Li J, Sandy J, Plaas A, Sumner DR. Initial application of EPIC-muCT to assess mouse articular cartilage morphology and composition: effects of aging and treadmill running. Osteoarthritis Cartilage. 2012;20(8):887–95. 22609479 10.1016/j.joca.2012.04.012 PMC3817026

[22] Mankin HJ, Dorfman H, Lippiello L, Zarins A. Biochemical and metabolic abnormalities in articular cartilage from osteo-arthritic human hips. J Bone Joint Surg. 1971;53(3):523–37. 5580011

[23] Pastoureau P, Leduc S, Chomel A, De Ceuninck F. Quantitative assessment of articular cartilage and subchondral bone histology in the meniscectomized guinea pig model of osteoarthritis. Osteoarthritis Cartilage. 2003;11(6):412–23. 12801481 10.1016/s1063-4584(03)00050-5

[24] Matas J, Orrego M, Amenabar D, Infante C, Tapia-Limonchi R, Cadiz MI, Alcayaga-Miranda F, González PL, Muse E, Khoury M, Figueroa FE, Espinoza F. Umbilical cord-derived mesenchymal stromal cells (MSCs) for knee osteoarthritis: repeated MSC dosing is superior to a single MSC dose and to hyaluronic acid in a controlled randomized phase I/II trial. Stem Cells Transl Med. 2019;8(3):215–24. 30592390 10.1002/sctm.18-0053 PMC6392367

[25] Simon D, Mascarenhas R, Saltzman BM, Rollins M, Bach BR, MacDonald P. The relationship between anterior cruciate ligament injury and osteoarthritis of the knee. Adv Orthop. 2015; 2015:928301. 10.1155/2015/928301 PMC4410751 25954533

[26] Weidow J, Pak J, Kärrholm J. Different patterns of cartilage wear in medial and lateral gonarthrosis. Acta Orthop Scand. 2002;73(3):326–9. 12143982 10.1080/000164702320155347

[27] Liu N, Fu D, Yang J, Liu P, Song X, Wang X, Li R, Fu Z, Chen J, Gong X, Chen C, Yang L. Asiatic acid attenuates hypertrophic and fibrotic differentiation of articular chondrocytes via AMPK/PI3K/AKT signaling pathway. Arthritis Res Ther. 2020;22(1):112. 32398124 10.1186/s13075-020-02193-0 PMC7218496

[28] Han T, Zhu T, Lu Y, Wang Q, Bian H, Chen J, Qiao L, He TC, Zheng Q. Collagen type X expression and chondrocyte hypertrophic differentiation during OA and OS development. Am J Cancer Res. 2024;14(4):1784–801. 38726262 10.62347/JWGW7377 PMC11076255

[29] Fuggle NR, Cooper C, Oreffo ROC, Price AJ, Kaux JF, Maheu E, Cutolo M, Honvo G, Conaghan PG, Berenbaum F, Branco J, Brandi ML, Cortet B, Veronese N, Kurth AA, Matijevic R, Roth R, Pelletier JP, Martel-Pelletier J, Vlaskovska M, Thomas T, Lems WF, Al-Daghri N, Bruyère O, Rizzoli R, Kanis JA, Reginster JY. Alternative and complementary therapies in osteoarthritis and cartilage repair. Aging Clin Exp Res. 2020;32(4):547–60. 32170710 10.1007/s40520-020-01515-1 PMC7170824

[30] Bendele AM. Animal models of osteoarthritis. J Musculoskel Neuron Interact. 2001;1(4):363–76. 15758487

[31] Lu L, Dai C, Zhang Z, Du H, Li S, Ye P, Fu Q, Zhang L, Wu X, Dong Y, Song Y, Zhao D, Pang Y, Bao C. Treatment of knee osteoarthritis with intraarticular injection of autologous adipose-derived mesenchymal progenitor cells: a prospective, randomized, double-blind, active-controlled, phase IIb clinical trial. Stem Cell Res Ther. 2019;10(1):143. 31113476 10.1186/s13287-019-1248-3 PMC6528322

[32] Lu L, Dai C, Du H, Li S, Ye P, Zhang L, Duffy X, Song Y, Togashi R, Vangsness CT, Bao C. Intra-articular injections of allogeneic human adipose-derived mesenchymal progenitor cells in patients with symptomatic bilateral knee osteoarthritis: a phase I pilot study. Regen Med. 2020;15(5):1625–36. 32677876 10.2217/rme-2019-0106

[33] Wyles CC, Houdek MT, Behfar A, Sierra R. Mesenchymal stem cell therapy for osteoarthritis: current perspectives. Stem Cells Cloning. 2015;28(8):117–24. 10.2147/SCCAA.S68073 PMC4559256 26357483

[34] Wang AT, Feng Y, Jia HH, Zhao M, Yu H. Application of mesenchymal stem cell therapy for the treatment of osteoarthritis of the knee: a concise review. World J Stem Cells. 2019;11(4):222–35. 31110603 10.4252/wjsc.v11.i4.222 PMC6503460

[35] Phinney GD, Pittenger MF. Concise Review: MSC-derived exosomes for cell-free therapy. Stem Cells. 2017;35(4):851–8. 28294454 10.1002/stem.2575

[36] Wakitani S, Imoto K, Kimura T, Ochi T, Matsumoto K, Nakamura T. Hepatocyte growth factor facilitates cartilage repair: full thickness articular cartilage defect studied in rabbit knees. Acta Orthop Scand. 1997;68(5):474–80. 9385250 10.3109/17453679708996266

[37] Stewart AA, Byron CR, Pondenis H, Stewart MC. Effect of fibroblast growth factor-2 on equine mesenchymal stem cell monolayer expansion and chondrogenesis. Am J Vet Res. 2007;68(9):941–5. 17764407 10.2460/ajvr.68.9.941

[38] Mizuno M, Katano H, Otabe K, Komori K, Matsumoto Y, Fujii S, Ozeki N, Tsuji K, Koga H, Muneta T, Matsuyama A, Sekiya I. Platelet-derived growth factor (PDGF)-AA/AB in human serum are potential indicators of the proliferative capacity of human synovial mesenchymal stem cells. Stem Cell Res Ther. 2015;10(6):243. 10.1186/s13287-015-0239-2 PMC4675012 26652649

[39] Park DS, Park JC, Lee JS, Kim TW, Kim KJ, Jung BJ, Shim EK, Choi EY, Cho KS, Kim CS. Effect of FGF-2 on collagen tissue regeneration by human vertebral bone marrow stem cells. Stem Cells Dev. 2015;24(2):228–43. 25122057 10.1089/scd.2014.0148

[40] Hwang OK, Noh YW, Hong JT, Lee JW. Hypoxia pretreatment promotes chondrocyte differentiation of human adipose-derived stem cells via vascular endothelial growth factor. Tissue Eng Regen Med. 2020;17(3):335–50. 32451775 10.1007/s13770-020-00265-5 PMC7260353

[41] Kitahashi T, Kogawa R, Nakamura K, Sekiya I. Integrin β1, PDGFRβ, and type II collagen are essential for meniscus regeneration by synovial mesenchymal stem cells in rats. Sci Rep. 2022;12(1):14148. 35986079 10.1038/s41598-022-18476-2 PMC9391488

[42] Le H, Xu W, Zhuang X, Chang F, Wang Y, Ding J. Mesenchymal stem cells for cartilage regeneration. J Tissue Eng. 2020;26(11):2041731420943839. 10.1177/2041731420943839 PMC7457700 32922718

[43] Chuma H, Mizuta H, Kudo S, Takagi K, Hiraki Y. One day exposure to FGF-2 was sufficient for the regenerative repair of full-thickness defects of articular cartilage in rabbits. Osteoarthritis Cartilage. 2004;12(10):834–42. 15450534 10.1016/j.joca.2004.07.003

[44] Mao G, Zhang Z, Hu S, Zhang Z, Chang Z, Huang Z, Liao W, Kang Y. Exosomes derived from miR-92a-3p-overexpressing human mesenchymal stem cells enhance chondrogenesis and suppress cartilage degradation via targeting WNT5A. Stem Cell Res Ther. 2018;9(1):247. 30257711 10.1186/s13287-018-1004-0 PMC6158854

[45] Heo JS, Kim S. Human adipose mesenchymal stem cells modulate inflammation and angiogenesis through exosomes. Sci Rep. 2022;12(1):2776. 35177768 10.1038/s41598-022-06824-1 PMC8854709

[46] Yoo KH, Thapa N, Chwae YJ, Yoon SH, Kim BJ, Lee JO, Jang YN, Kim J. Transforming growth factor–β family and stem cell–derived exosome therapeutic treatment in osteoarthritis. Int J Mol Med. 2022;49(5):62. 35293597 10.3892/ijmm.2022.5118 PMC8930092

[47] Wang J, Chen ZJ, Zhang ZY, Shen MP, Zhao B, Zhang W, Zhang Y, Lei JG, Ren CJ, Chang J, Xu CL, Li M, Pi YY, Lu TL, Dai CX, Li SK, Li P. Manufacturing, quality control, and GLP-grade preclinical study of nebulized allogenic adipose mesenchymal stromal cells-derived extracellular vesicles. Stem Cell Res Ther. 2024;15(1):95. 38566259 10.1186/s13287-024-03708-1 PMC10988864

[48] Chiang ER, Ma HL, Wang JP, Liu CL, Chen TH, Huang SC. Allogeneic mesenchymal stem cells in combination with hyaluronic acid for the treatment of osteoarthritis in rabbits. PLoS ONE. 2016;11(2):e0149835. 26915044 10.1371/journal.pone.0149835 PMC4767225

[49] Lamo-Espinosa JM, Mora G, Blanco JF, Granero-Moltó F, Nuñez-Córdoba JM, Sánchez-Echenique C, Bondía JM, Aquerreta JD, Andreu EJ, Ornilla E, Villarón EM, Valentí-Azcárate A, Sánchez-Guijo F, Del Cañizo MC, Valentí-Nin JR, Prósper F. Intra-articular injection of two different doses of autologous bone marrow mesenchymal stem cells versus hyaluronic acid in the treatment of knee osteoarthritis: Multicenter randomized controlled clinical trial (phase I/II). J Transl Med. 2016;14(1):246. 27565858 10.1186/s12967-016-0998-2 PMC5002157

[50] Bastos R, Mathias M, Andrade R, Bastos R, Balduino A, Schott V, Rodeo S, Espregueira-Mendes J. Intra-articular injections of expanded mesenchymal stem cells with and without addition of platelet-rich plasma are safe and effective for knee osteoarthritis. Knee Surg Sports Traumatol Arthrosc. 2018;26(11):3342–50. 29511819 10.1007/s00167-018-4883-9

[51] Huang H, Zhang P, Xiang C, Zeng C, Du Q, Huang W. Effect of bone marrow mesenchymal stem cell transplantation combined with lugua polypeptide injection on osteoarthritis in rabbit knee joint. Connect Tissue Res. 2022;63(4):370–81. 34355626 10.1080/03008207.2021.1962314

[52] Feng C, Luo X, He N, Xia H, Lv X, Zhang X, Li D, Wang F, He J, Zhang L, Lin X, Lin L, Yin H, He J, Wang J, Cao W, Wang R, Zhou G, Wang W. Efficacy and persistence of allogeneic adipose-derived mesenchymal stem cells combined with hyaluronic acid in osteoarthritis after intra-articular injection in a sheep model. Tissue Eng Part A. 2018;24(3–4):219–33. 28486025 10.1089/ten.TEA.2017.0039

[53] Wei P, Bao R. Intra-articular mesenchymal stem cell injection for knee osteoarthritis: mechanisms and clinical evidence. Int J Mol Sci. 2023;24(1):59. 10.3390/ijms24010059 PMC9819973 36613502

