# Supplementary material for: Therapeutic efficacy of intra-articular injection of human adipose-derived mesenchymal stem cells in a sheep model of knee osteoarthritis
Source: Stem Cell Res Ther. 2025 Jan 23;16:24. doi: 10.1186/s13287-025-04143-6 (PMC11755983; doi:10.1186/s13287-025-04143-6)
Supplement: Supplementary file 1 — Supplementary Material 1: Table 1. Quantification of growth factors in the conditioned medium of haMSCs (in pg/mL). [file 13287_2025_4143_MOESM1_ESM.docx]

Supplementary Table 1. Quantification of growth factors in the conditioned medium of haMSCs (in pg/mL).

| **Group** | **VEGF** | **PDGF** | **HGF** | **FGF-2** |
| --- | --- | --- | --- | --- |
| Control | 5.24 | 1.96 | 3.63 | 10.09 |
| haMSCs | 1856.00±475.34 | 93.00±1.48 | 90.40±3.54 | 53.56±0.57 |

Abbreviations: haMSCs, human adipose-derived mesenchymal stem cells; VEGF, vascular endothelial growth factor; PDGF, platelet-derived growth factor; HGF, hepatocyte growth factor; FGF, fibroblast growth factor.
